# Effects of *Fragaria vesca* leaf extract on the initial pellicle—an atypical representative among polyphenol-containing substances

**DOI:** 10.3389/froh.2026.1744798

**Published:** 2026-04-10

**Authors:** Jola Frey, João Victor Frazão Câmara, Johanna Dudek, Matthias Hannig, Simone Trautmann

**Affiliations:** Department of Operative Dentistry, Periodontology and Preventive Dentistry, Saarland University, Homburg/Saar, Germany

**Keywords:** atomic absorption spectrometry, dental pellicle, enamel demineralization, polyphenols, transmission electron microscopy

## Abstract

**Objective:**

Polyphenol-containing mouth rinses are known to improve the protective properties of the dental pellicle against acids by increasing its thickness and density. Even though *fragaria vesca* (wild strawberry) leaf extract (WSLE) contains high concentrations of polyphenols and possesses beneficial properties on the oral biofilm, its effects on dental pellicle have not been studied yet.

**Methods:**

Transmission electron microscopy (TEM) and atomic adsorption spectrometry (AAS) were used to visualize the ultrastructure of the 6-min and 120-min pellicle as well as measure the calcium release of the enamel surface after rinsing for 1 min with WSLE or water as control and applying different acidic challenges (1% citric acid, 0.01 M HCl or water).

**Results:**

Mouth rinsing with WSLE did neither increase thickness or density of the pellicle nor improve its protectivity against acids. Compared to water-rinsing, samples showed equal or even higher dissolution of the pellicle upon acidic attack. AAS experiments demonstrated an increased calcium release and the leaf extract was shown to possess an appreciable calcium content.

**Conclusion:**

WSLE represents an outlier in the group of polyphenol-containing mouth rinses in terms of its effects on the dental pellicle. The increased calcium release upon rinsing and its appreciable calcium content suggests it may act as a calcium source for the dental pellicle.

**Clinical significance:**

Based on its previously demonstrated anti-adhesive properties on the oral biofilm and the current results on its remarkable calcium content, WSLE presents as a promising candidate for daily mouth rinse use, warranting further clinical studies.

## Introduction

Polyphenols, a diverse group of plant-derived secondary metabolites, have gained increasing attention in biomedical research due to their antioxidant, anti-inflammatory, and antimicrobial properties (1, 2). They are defined as molecules containing at least one aromatic ring with one or more hydroxyl groups in addition to different substituents (3). Their potential role in oral health, particularly in the prevention of dental diseases, has become a focal point of recent investigations (4–6).

The acquired enamel pellicle—a proteinaceous layer formed by the continuous adsorption of salivary components to all orally exposed surfaces—acts as a diffusion barrier, protects the underlying dental surfaces against chemical and mechanical damages and represents the base for bacterial adhesion and oral biofilm development (7, 8). Modifying the pellicle to enhance its protective properties, the so-called “pellicle engineering”, offers a promising strategy for preventive dentistry.

The ingestion of polyphenol-containing foods and beverages is typically accompanied by the astringency phenomenon, a mouth puckering combined with blunt teeth sensation (9, 10). This is based on the fact, that polyphenols interact with salivary and pellicle proteins, leading to protein aggregations and structural changes in the pellicle (5, 11, 12). The polyphenol-protein-interactions are mainly relying on hydrogen bonding and hydrophobic interactions (3). These modifications typically increase the pellicle thickness and density (4, 13–16), reduce receptor sites for bacterial adhesion (5, 11, 17, 18), and enhance its resistance to acidic challenges (11, 15, 16, 19–25). In this context, citric acid as a nutritionally derived extrinsic acid and hydrochloric acid associated with gastric reflux as an intrinsic acid must be considered common risk factors for erosive challenges. The concept of pellicle engineering has emerged as a widespread approach in preventive dentistry (26–29). Polyphenols are particularly suitable for this purpose due to their natural origin, biocompatibility, and multifunctional properties. There is one study analyzing the effects of *fragaria vesca* (wild strawberry) leaf extract (WSLE) mouth rinses on the oral biofilm (30). The authors chose *fragaria vesca* leaves from the list of official tea drugs of the German pharmacy index (31) based on missing data on its effects on the oral biofilm and its multiple and high polyphenol content (5%–10%), including several ellagitannins, flavonols and proanthocyanidins as active substances (30, 32). The results showed a significant reduction of initial bacterial colonization, a decrease in glucan formation as well as a thicker and more electron-dense layer of the 8 h pellicle upon an initial mouth rinse with WSLE for 10 min.

Current research highlights various positive effects of polyphenol-containing applications on oral health. *Fragaria vesca* exhibits a high polyphenol content and was so far only investigated in a single study with promising results, making it highly reasonable to investigate its effects on the dental pellicle in more detail. Therefore, the aim of the current study was to elucidate the effects of WSLE-mouth rinsing on the ultrastructure and protective properties of the initial pellicle.

## Materials and methods

### Human subjects

*In situ*-pellicles were formed by three subjects (aged 21–31, one female, two male), all members of the laboratory staff or students. They were all non-smokers and showed no signs of caries or periodontitis in the last two years, exhibited no general disease and no intake of medication. An experienced dentist carried out visual oral examination. Prior to the start of the study, they had given informed, written consent to participate. Pellicle collection protocols were approved by the medical ethic committee of the Medical Association of Saarland, Germany (proposal #54/21, 2021).

To avoid circadian effects on the salivary composition, all experiments started at 8 am. 2 h before starting, the subjects refrained from any food or beverages and conducted individual oral hygiene. 30 min before pellicle formation, subjects used dental floss and brushed teeth without toothpaste to avoid influences caused by any of its ingredients.

### Specimens preparation

**Figure 1:**
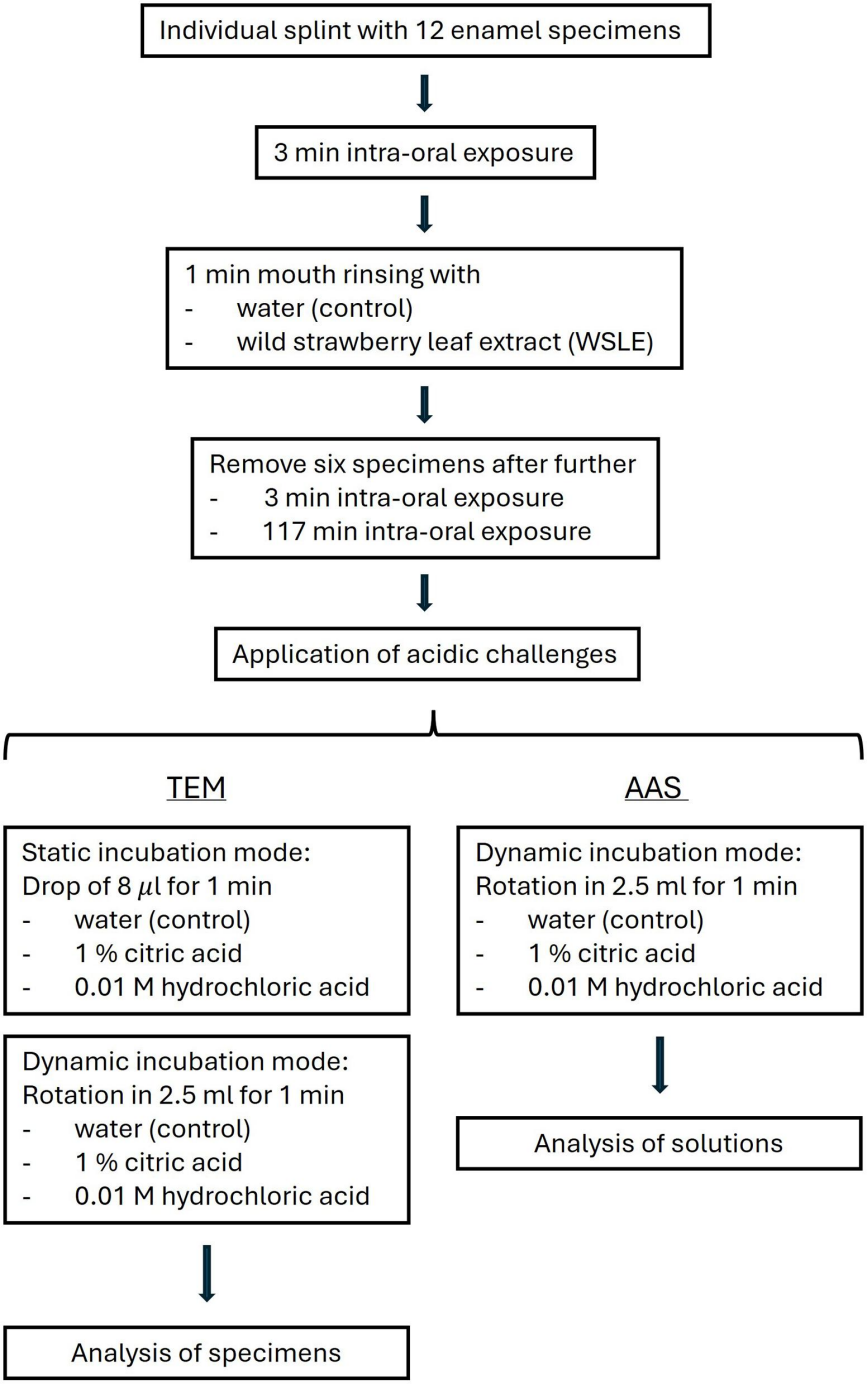
Flowchart depicting single experimental steps conducted individually with samples of each of three subjects.

Enamel slabs were derived from the facial surfaces of bovine incisors of two years old cattle (tested bovine spongiform encephalopathy (BSE)-negative). The surfaces of the specimens were polished with abrasive paper and increasing grit sizes (120–4,000 grit) by wet grinding. Specimens were sterilized and purified from any polishing residues with 3% NaOCl, washed with water, ultrasonicated in 70% isopropanol, air dried and rehydrated in sterile water for minimum 12 h. Individual splints containing a number of 12 fixed enamel specimens per rinsing solution (water or WSLE) and per analytical method (transmission electron microscopy (TEM) or atomic adsorption spectrometry (AAS)) were analysed. Additionally, six specimens were used in parallel as controls without intra-oral exposure. Given the number of three subjects within the study, a total number of 144 (3 × 48) specimens plus six controls were analysed. Figure 1 depicts an overview of the experimental steps.

### Preparation of wild strawberry leaf extract (WSLE)

WSLE was prepared similar to that described by Kirsch et al. 2020 (30). Briefly, 3% w/v ground *fragaria vesca* (wild strawberry) leaves (Kräuter Schulte, Germany) were homogenized in water (Ampuwa, Fresenius, Germany) by vortexing, followed by an extraction via ultrasonication for 30 min. After centrifugation, the polyphenol-containing supernatant was saved and the pellet was extracted a second time using the same procedure. After pooling both supernatants and adjusting to the final volume with water, the extract was filter sterilized. It exhibited a stable pH of 5.5 (for 3 weeks) and was stored at 4 °C for a maximum of 7 days. An extract from green tea (Sencha) (Kräuter Schulte, Germany) was prepared in parallel, following the same protocol. Both extracts were sent to Eurofins Dr. Specht International GmbH (Hamburg, Germany) to determine the polyphenol content by ultraviolet–visible (UV-VIS) spectrophotometry and calcium content by inductively coupled plasma mass spectrometry (ICP-MS).

### *In situ*-pellicle formation

Individual splints containing fixed enamel specimens were exposed to the oral cavity for 3 min. Then, subjects rinsed with 10 ml water for 1 min. After additional 3 min of pellicle formation, the splints were removed to detach the 6-min pellicle samples. Specimens were rinsed with water to eliminate any salivary residues and subjected to acidic challenges. Splints containing residual specimens were reinserted in the oral cavity and pellicle was allowed to form for further 117 min. After removal of the 120 min pellicle samples, specimens were rinsed with water and subjected to acidic challenges. Subsequently, the complete procedure was repeated using WSLE as mouth rinsing solution.

### Acidic challenges

For transmission electron microscopy analyses, a static and a dynamic incubation mode were used for etching pellicle samples with different test solutions. The two incubation modes were used to analyse both the effect of laboratory *in vitro*-conditions (static incubation) and a more physiological scenario with shearing forces and continuous movement present in the oral cavity (dynamic mode). Based on previous test results, only the dynamic incubation mode was applied for atomic absorption spectrometry. The static mode was performed by pipetting a drop (8 µL) of the test solution (1% citric acid with pH 2.5, 0.01 M HCl with pH 2.2 or water as control), on the enamel surface. After 1 min, the drop was washed away with water. For dynamic mode etching, the specimens were placed in tubes (Eppendorf, Germany), filled with 2.5 mL 1% citric acid, 0.01 M HCl or water. Tubes were rotated at 35 rpm and a 30° angle for 1 min (loopster digital, Ika, Germany). Finally, the specimens were removed and subjected to transmission electron microscopy. For atomic absorption spectrometry, the incubation solutions were replenished with additional 2.5 mL of the respective solution for a final sample volume of 5 mL.

### Transmission electron microscopy (TEM)

Enamel specimens were incubated in fixing solution (1% glutaraldehyde, 1% paraformaldehyde, cacodylate buffer: 0.1 M cacodylate/HCl pH 7.5) for 1 h at 4 °C, washed in cacodylate buffer, and post-fixed in 1% osmium tetroxide for 1 h. Subsequently, specimens were dehydrated in an ascending series of ethanol concentrations and embedded in Araldite CY212 (Agar Scientific, Stansted, UK). After removing the enamel part by decalcification in 1 M HCl for at least 3 h, the specimens were reembedded in Araldite. Ultrathin sections were cut with an ultramicrotome (Ultracut E, Bensheim, Germany) using a diamond knife (Microstar 45°, Plano GmbH, Wetzlar, Germany). Sections were mounted on pioloform-coated copper grids (Plano, Wetzlar, Germany), contrasted with 2% uranyl acetate for 10 min and lead citrate (pH 11.95) for 6 min and analysed by a TECNAI 12 BioTwin (FEI, Eindhoven, Netherlands).

### Atomic absorption spectrometry (AAS)

To measure solely the calcium release from the enamel surface, all residual parts of the specimens were covered with nail polish (Maybelline New York, New York, USA) before intraoral exposure and acidic challenges. The enamel surface areas of specimens were measured with a digital caliper for later calculation of calcium release. Samples were mixed with lanthanum chloride to eliminate interference of other ions, diluted 1:10 and atomized using a flame atomizer. Calcium release was measured in triplicates at 422 nm using atomic absorption spectrometry (ContrAA 700, Analytik Jena, Germany; software Aspect CS 2.2.1.0) and normalized to dilution factor and surface area of specimens, expressed in µg/mm^2^. Data interpretation was based on transparent descriptive statistics (means and standard deviations).

## Results

In the present study, the ultrastructure of the pellicle and the calcium release from the pellicle-covered enamel surface were analysed after application of wild strawberry leaf extract (WSLE) or water (control) as mouth rinsing solution and exposure to different acidic challenges. To determine the concentration of polyphenols as active constituents of the WSLE, an aliquot of the extract was analysed by Eurofins Dr. Specht International GmbH (Hamburg, Germany). The analysis revealed polyphenol concentrations ranging from 499 (± 128) mg/l, calculated as epicatechin equivalent, to 1190 (± 293) mg/l, calculated as epigallocatechin gallate (EGCG) equivalent (Table 1). For comparison with a well-studied polyphenol-rich substance, an extract of green tea was analysed in parallel and found to contain approximately three times the polyphenol content of the WSLE for the single polyphenol equivalents (data not shown).

**Table 1:** List of polyphenol content measured by UV-VIS spectrophotometry, provided as concentration (mg/L) determined in the WSLE as equivalents of different polyphenols. The limit of detection is listed for each test parameter in mg/L.

Test parameter	Limit of detection (mg/L)	Concentration (mg/L)
Polyphenols calculated as gallic acid equivalent	100	822 ± 206
Polyphenols calculated as tannic acid equivalent	120	990 ± 245
Polyphenols calculated as catechin equivalent	80	656 ± 166
Polyphenols calculated as epicatechin equivalent	60	499 ± 128
Polyphenols calculated as EGCG equivalent	150	1190 ± 293
Polyphenols calculated as caffeic acid equivalent	93	762 ± 191
Polyphenols expressed as pyrogallol equivalent	81	663 ± 168

**Figure 2:**
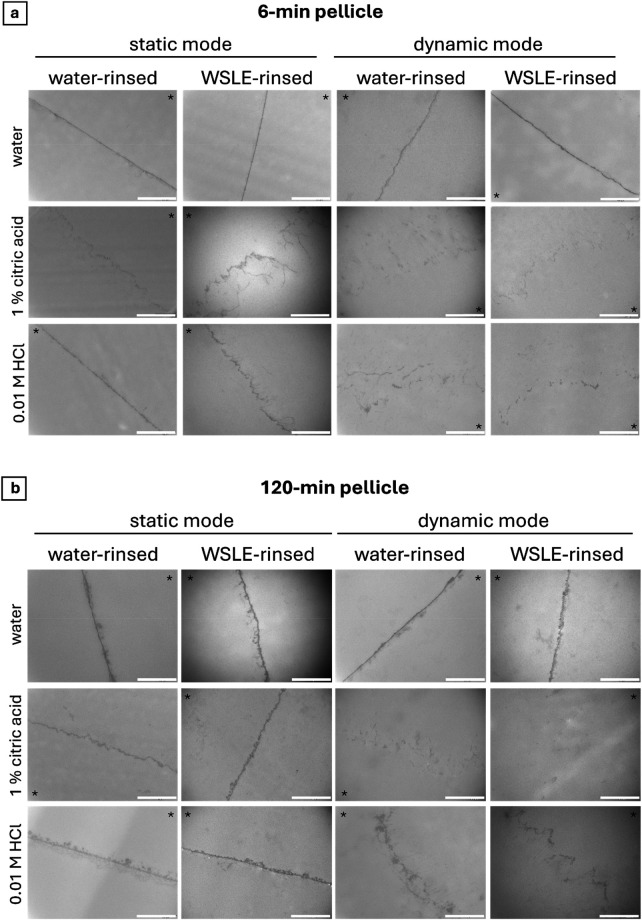
Representative transmission electron microscopy images depicting 6 min **(a)** or 120 min **(b)** pellicle formed on dental enamel. After 3 min of pellicle formation, oral cavity was rinsed with water or wild strawberry leaf extract (WSLE) for 1 min. After pellicle formation was completed by additional 3 or 117 min oral exposure, samples were etched under static or dynamic incubation modes with water (control), 1% citric acid or 0.01 M HCl for 1 min. Asterisks mark the pellicle side, original magnification: x 49.000, bars 1 µm.

To visualize potential ultrastructural changes in the initial pellicle layer and the dental surface, comparisons between pellicles formed on enamel specimens rinsed with WSLE or water as control were analysed after different acidic challenges by transmission electron microscopy (TEM). Thereby, both the 6-min and 120-min pellicle layers did not show differences in pellicle thickness after the application of WSLE rinsing solution in comparison to water as indicated by the analogous pellicle thickness and electron density of the layer (Figures 2a,b upper rows). Rinsing with WSLE resulted in a slightly pronounced dissolution of the 6 min pellicle layer and deeper infiltrations upon static etching with 1% citric acid and 0.01 M HCl. Under dynamic mode-conditions, both rinsing-solutions showed similar, almost completely dissolved, deeply infiltrated areas after acidic treatment shown by the fully disintegrated pellicle layer and its deep infiltrative extensions into the previous enamel surface (Figure 2a middle and lower rows). The etching effects between samples rinsed with WSLE or water were comparable between the 120 min pellicle samples under static conditions, whereat 1% citric acid and, to a lower extent, 0.01 M HCl in dynamic mode resulted in a stronger dissolution of the WSLE-rinsed pellicle compared to the water-rinsed control (Figure 2b middle and lower rows).

**Figure 3:**
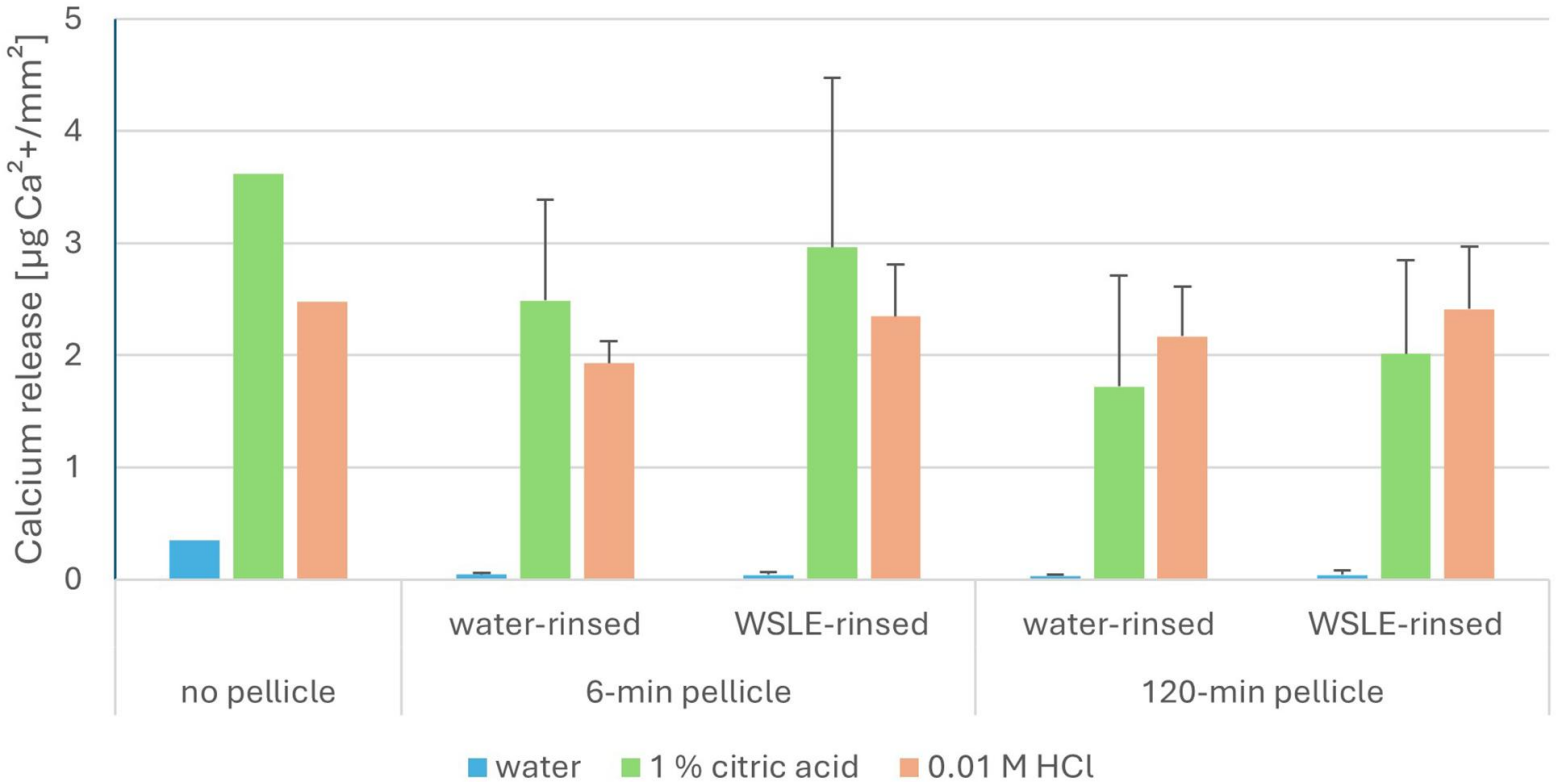
Atomic absorption spectrometry-measurements of calcium release [µg Ca^2+^/mm²] from enamel surfaces without (no pellicle), covered with 6 min or 120 min pellicle layer, averaged over three subjects. Bars represent mean values of calcium release, error bars indicate standard deviation (SD) of the measurements. In case of the 6 min or 120 min pellicle samples, oral cavity was rinsed after 3 min of pellicle formation with water (as control) or wild strawberry leaf extract (WSLE) for 1 min. After pellicle formation was completed by additional 3 or 117 min oral exposure, samples were etched under dynamic conditions with water as control, 1% citric acid or 0.01 M HCl for 1 min.

To evaluate potential protective effects of the WSLE-rinsing, the calcium release of pellicle covered enamel specimens was measured via atomic absorption spectroscopy (AAS) after application of WSLE or water as rinsing solution followed by different acidic challenges. Figure 3 depicts the results of the calcium release measurements averaged over three subjects, individual results are shown in the supplement (supplemental Figure S1). Thereby, compared to the no pellicle control, a reduced calcium release was measured in the presence of the 6 min and 120 min pellicle in the samples etched with the water control or 1% citric acid. The application of 0.01 M HCl resulted in a lowered calcium release in the presence of the 6 min or 120 min pellicle after rinsing with water, whereas the preceding rinse with WSLE showed hardly no changes in calcium release in the presence of the pellicle, compared to the no pellicle-control. In case of rinsing with water and etching with 1% citric acid, a distinctly lowered calcium release of 31% or 52% was observed in the presence of the 6-min or 120-min pellicle layer compared to the control without pellicle. Upon water rinsing and etching with the 0.01 M HCl, a lowered calcium release of 22% or 12% was detected for the 6-min or 120-min pellicle samples. Rinsing with WSLE resulted in appreciably elevated calcium release levels compared to water-rinsing. Thereby, an increase of 19% or 17% was observed in the presence of the 6 min or 120 min pellicle upon etching with 1% citric acid. An increase of 22% or 11% in the presence of the 6-min or 120-min pellicle upon etching with 0.01 M HCl was detected. The individual differences between the three volunteers resulted in standard deviations between ±0.01 to 1.51.

Based on the increased calcium releases after rinsing with WSLE, an aliquot of the extract and an aliquot of green tea extract (GTE) were analysed by Eurofins Dr. Specht International GmbH (Hamburg, Germany) to determine the calcium content. This resulted in 93.0 (±19.0) mg/L calcium in the WSLE and 5.1 (±4.1) mg/L calcium in the GTE.

## Discussion

Transmission electron microscopy (TEM) and atomic absorption spectrometry (AAS) were demonstrated in previous studies to represent effective methods for assessing ultrastructural changes and calcium release of the pellicle and underlying dental surfaces (23, 33). The combination of both techniques currently represents the most suitable approach for evaluating the protective properties of the pellicle against acids. Due to the severely limited availability of healthy human teeth for experimental purposes, dental studies are predominantly conducted with alternative substitute materials. In this context, bovine dental hard tissue is the most frequently used alternative. Based on its close similarity to human enamel in structure, composition, and hardness, as well as its comparable outcomes in erosion behaviour and biofilm development in microbial caries models, bovine enamel has been widely established as a reliable substitute in dental research for many decades (34–38). In addition, UV-VIS spectrophotometry was used to measure the polyphenol content as active substances of the WSLE and GTE. The well-studied GTE represents a polyphenol-rich substance with manifold health-promoting aspects (11, 18, 39–41). Even though the measured polyphenol content of the WSLE represents only one third of the content in green tea, WSLE exhibits a comparatively high and noteworthy polyphenol content and was expected to induce a thickening of the pellicle and enhance its protective properties similar to other polyphenol-containing substances. For example, in a study of Lee et al., the polyphenol content of a 2% black tea extract was determined, ranging from 16 mg/l for epicatechin to 74 mg/l for EGCG (42). Even though the polyphenol content of this black tea extract was noticeably lower than the one of the WSLE, protective effects of black tea rinses as well as pellicle thickening effects were demonstrated in several studies (13, 43).

The erosive effects clearly visible in TEM-micrographs upon etching with 1% citric acid and 0.01 M HCl in most samples, being evident from a dissolution of the pellicle layer and infiltrations of most likely pellicle proteins in emerging cavities on the enamel surface, were stronger under dynamic compared to static conditions. This is most probably based on the prevalent shearing forces combined with higher acid volumes and induced mixing under dynamic conditions. In general, the dynamic mode more accurately reflects the physiological conditions of the oral cavity. To the best of our knowledge, the present study is the first to apply and comparatively investigate static and dynamic conditions for evaluating the erosion-protective properties of the dental pellicle upon rinsing with a polyphenol-containing mouth rinse. The observed differences between the samples etched with the static or dynamic mode highlight the importance of selecting preferably physiologically relevant conditions.

The differences in the erosive effect extent between etching with 1% citric acid or 0.01 M hydrochloric acid can be ascribed to the acid strengths and their respective concentrations. 1% citric acid was chosen as classical extrinsic erosion factor to mimic the consumption of acidic beverages (44). The 0.01 M HCl was used as example of an intrinsic stimulus, gastric acid, which is mainly composed of hydrochloric acid (45).

As polyphenols were manifold shown to enhance the protective properties of the pellicle by increasing its thickness and density (4, 13–16), similar effects would have been expected on the initial pellicle upon rinsing with wild strawberry leaf extract (WSLE). The observed missing increase of the 6 min and 120 min pellicle contradicts these common observations. In a study of Kirsch et al., rinsing with WSLE was shown to clearly increase the thickness of the 8 h pellicle on dental enamel (30). However, in that study the subjects rinsed for 10 min, representing a 10-fold elongation of the current rinsing time. The present 1-min rinsing was chosen in terms of an application-oriented use for a mouth rinse and might explain the observed differences in the thickening effect based on maximized protein aggregation and incorporation of polyphenols in the pellicle layer upon the 10-fold increased rinsing period. Additionally, compared to the water control, rinsing with WSLE resulted in a pronounced dissolution of the pellicle layer and even deeper infiltrations in the enamel surface upon acidic attack under several conditions. These results are not in line with the so far published data on the effects of polyphenol-containing mouth rinses on the dental pellicle and point to a rather reduced protectivity of the initial pellicle upon application of WSLE (11, 15, 16, 19–25).

The AAS data were interpreted using transparent descriptive statistics based on means and standard deviations. The high standard deviations originate from individual differences between the subjects. These differences in calcium release are probably attributable to the highly individualized composition of the pellicle proteome, which has been described as possessing an individual fingerprint and, accordingly, most likely distinct protective properties against acids (46, 47). Despite the considerable inter-individual variability, all subjects exhibited the same overall trend: an increase in calcium release upon rinsing with WSLE.

The reduced calcium release detected in the presence of the 6-min and 120 min pellicle, compared to the “no pellicle”-control, are in line with the manifold described pellicle function of a protective barrier against chemical damages (8, 48, 49). The increased calcium release after rinsing with WSLE compared to water under all conditions pointed to a reduced protectivity of the pellicle and/or a notable calcium content of the leaf extract combined with its incorporation in the pellicle even after the application-oriented use of 1 min rinses. These observations are not in line with the so far published data on a reduced calcium release upon the application of different polyphenol-containing mouth rinses (15, 16, 23, 50) and led to calcium measurements of the extract, demonstrating an appreciable calcium content. In the likewise analysed polyphenol-rich GTE, an approximately 19-fold lower calcium content was measured, highlighting the exceptional calcium content of the WSLE.

The shown increased demineralization of the enamel surface in case of the 6-min pellicle samples rinsed with WSLE and etched with 1% citric acid or 0.01 M HCl in the static mode could explain the increased calcium releases measured during AAS-experiments. However, the AAS experiments were conducted solely under dynamic mode conditions, among which no increased demineralization/deeper micro cavities in the enamel surfaces rinsed with WSLE were detected by TEM-analyses. Therefore, the currently measured increase in calcium released after rinsing with WSLE might also be due to an incorporation of the calcium-containing WSLE in the pellicle and its release upon etching. This scenario would be in line with the proposed calcium reservoir function of the pellicle (8).

## Conclusion

The findings demonstrate WSLE as outlier in the group of polyphenol-containing mouth rinses and their effects on the dental pellicle. Even though *fragaria vesca* possesses a high polyphenol content and was shown to reduce bacterial adhesion in the oral biofilm, no beneficial effects were observed in terms of an increased resistance of the initial pellicle against acids upon rinsing with its leaf extract. WSLE’s notable calcium content suggests it may act as a calcium source for the dental pellicle. In summary, and in light of its previously demonstrated anti-adhesive properties, WSLE presents as a promising candidate for daily mouth rinse use, warranting further clinical studies.

## Data Availability

The raw data supporting the conclusions of this article will be made available by the authors, without undue reservation.
