# Supplementary figures and images for: Effects of Fragaria vesca leaf extract on the initial pellicle—an atypical representative among polyphenol-containing substances
Source: Front Oral Health. 2026 Apr 10;7:1744798. doi: 10.3389/froh.2026.1744798 (PMC13106438; doi:10.3389/froh.2026.1744798)

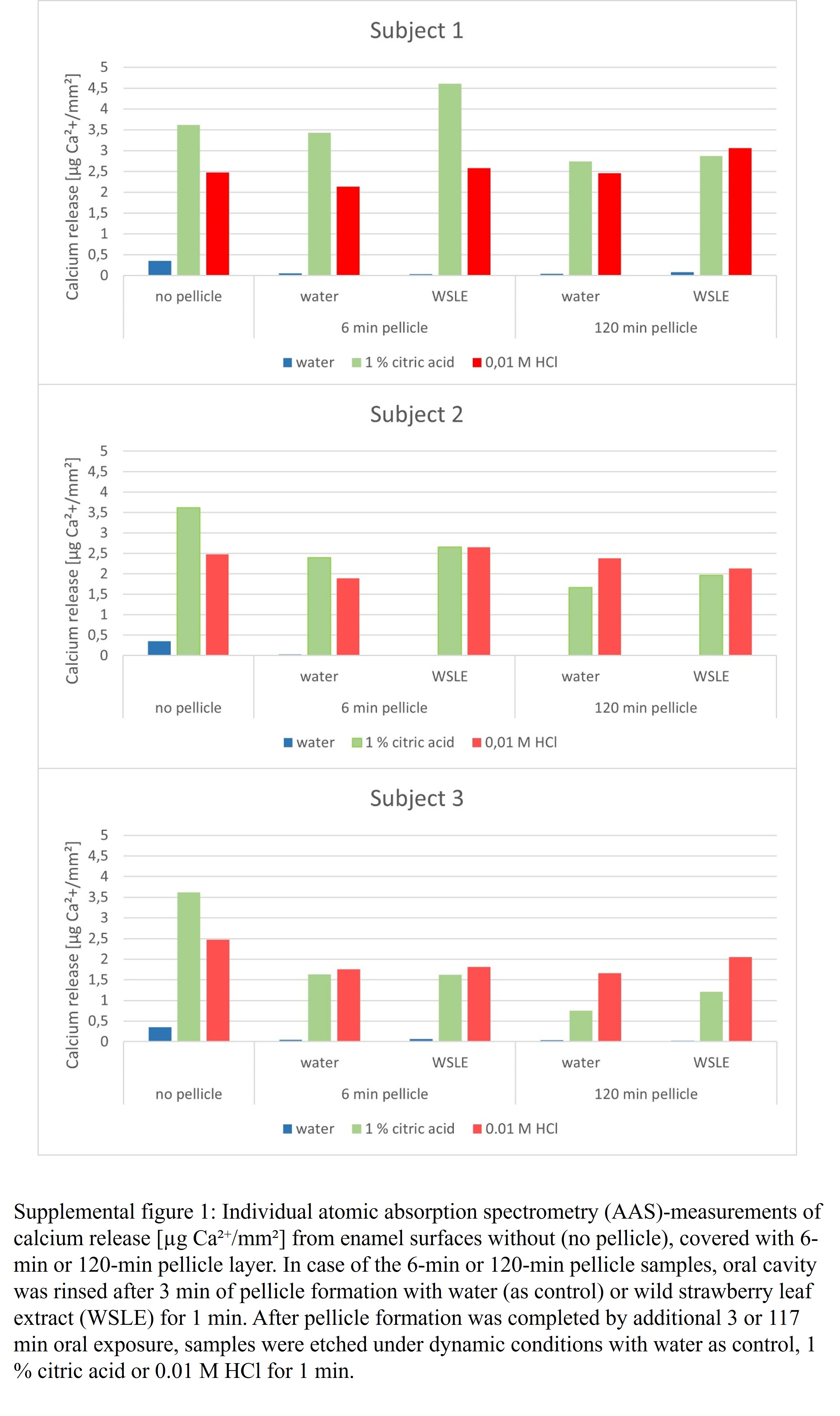

Supplement: Supplementary file 1 [file Image1.jpeg]
